# Supplementary material for: High-throughput multivariable Mendelian randomization analysis prioritizes apolipoprotein B as key lipid risk factor for coronary artery disease
Source: Int J Epidemiol. 2020 Jan 11;50(3):893–901. doi: 10.1093/ije/dyaa216 (PMC8271202; doi:10.1093/ije/dyaa216)
Supplement: dyaa216_Supplementary_Data [file dyaa216_supplementary_data.pdf]

Supplementary file: High-throughput multivariable Mendelian randomization analysis prioritizes apolipoprotein B as key lipid risk factor for coronary artery disease

Verena Zuber[1,2], Dipender Gill[1], Mika Ala-Korpela[3,4], Claudia Langenberg[5], Adam Butterworth[6,7,8,9,10,11], Leonardo Bottolo[12,13,2], and Stephen Burgess[2,6,*]

[1]Department of Epidemiology and Biostatistics, School of Public Health, Imperial College London, London, UK
[2]MRC Biostatistics Unit, School of Clinical Medicine, University of Cambridge, Cambridge, UK
[3]Computational Medicine, Faculty of Medicine, University of Oulu & Biocenter Oulu, Oulu, Finland
[4]NMR Metabolomics Laboratory, School of Pharmacy, University of Eastern Finland, Kuopio, Finland
[5]MRC Epidemiology Unit, School of Clinical Medicine, University of Cambridge, Cambridge, UK
[6]British Heart Foundation Cardiovascular Epidemiology Unit, Department of Public Health and Primary Care, University of Cambridge, Cambridge, UK
[7]British Heart Foundation Centre of Research Excellence, University of Cambridge, Cambridge, UK
[8]National Institute for Health Research Blood and Transplant Research Unit in Donor Health and Genomics, University of Cambridge, Cambridge, UK
[9]National Institute for Health Research Cambridge Biomedical Research Centre, University of Cambridge and Cambridge University Hospitals, Cambridge, UK
[10]Health Data Research UK Cambridge, Wellcome Genome Campus and University of Cambridge, Cambridge, UK
[11]Department of Human Genetics, Wellcome Sanger Institute, Hinxton, UK
[12]Department of Medical Genetics, School of Clinical Medicine, University of Cambridge, Cambridge, UK
[13]The Alan Turing Institute, London, UK
[*]Corresponding author: Stephen Burgess sb452@medschl.cam.ac.uk; MRC

Biostatistics Unit, University of Cambridge, Cambridge CB2 0SR, UK

September 24, 2020

List of Tables

S1 List of lipoprotein and metabolite measurements included in the analyses. . . . S2
S2 Study summary table . . . S3
S3 Cook's distance to detect influential genetic variants . . . S4
S4 q-statistic to detect outlying genetic variants . . . S5
S5 Analysis including all genetic variants . . . S6
S6 Supplementary analysis 1 . . . S7
S7 Supplementary analysis 2 . . . S8
S8 Supplementary analysis 3 . . . S9
S9 Sensitivity analysis . . . S10
S10 Parameter check: Prior variance . . . S11
S11 Parameter check: Prior probability . . . S12

List of Figures

S1 Genetic correlation between lipoprotein measures and metabolites . . . S13
S2 Diagnostic plots with Cooks distance . . . S14
S3 Diagnostic plots with q-statistic . . . S15

Supplementary Tables

Abbreviation	Lipoprotein and metabolite measurements included
XXL.VLDL.TG	Triglyceride content in chylomicrons and extra-extra large VLDL
XL.VLDL.TG	Triglyceride content in extra-large VLDL
L.VLDL.TG	Triglyceride content in large VLDL
M.VLDL.TG	Triglyceride content in medium VLDL
S.VLDL.TG	Triglyceride content in small VLDL
XS.VLDL.TG	Triglyceride content in extra-small VLDL
IDL.TG	Triglyceride content in IDL
XL.HDL.TG	Triglyceride content in extra-large HDL
S.HDL.TG	Triglyceride content in small HDL
Serum.TG	Serum total triglycerides
L.VLDL.C	Cholesterol content in large VLDL
M.VLDL.C	Cholesterol content in medium VLDL
S.VLDL.C	Cholesterol content in small VLDL
LDL.C	Cholesterol content in LDL
S.LDL.C	Cholesterol content in small LDL
IDL.C	Cholesterol content in IDL
XL.HDL.C	Cholesterol content in extra-large HDL
L.HDL.C	Cholesterol content in large HDL
M.HDL.C	Cholesterol content in medium HDL
HDL.C	Cholesterol content in HDL
Est.C	Esterified cholesterol
Serum.C	Serum total cholesterol
VLDL.D	VLDL diameter
LDL.D	LDL diameter
HDL.D	HDL diameter
ApoA1	Apolipoprotein A1
ApoB	Apolipoprotein B
SM	Sphingomyelins
Tot.FA	Total fatty acids
Tot.PG	Total phosphoglycerides

Table S1: List of lipoprotein and metabolite measurements included in the analyses.

	First author	Year	Pubmed	n	Cases	Controls	Study names (population)
Risk factor							
	Kettunen	2016	27005778	24,925			NMR GWAS (European, predominantly Finnish)
Outcome							
Main	Nelson	2017	28714975	453,595	113,937	339,658	CARDIoGRAMplusC4D (European, South&East Asian) and UK Biobank (European)
Sensitivity	Nikpay	2015	26343387	184,305	60,801	123,504	CARDIoGRAMplusC4D (European, South&East Asian)
	UK Biobank (European)	2019	31756303	367,703	29,278	338,425	UK Biobank (European)

Table S2: Study summary table

CARDIoGRAMplusC4D and UK Biobank

	rs	gene region	$Cd1$	$Cd2$	$Cd3$	max Cd
1	rs10903129	TMEM57	0.108	0.054	0.018	0.108
2	rs2923084	AMPD3	0.049	0.069	0.068	0.069
3	rs6489818	MAPKAPK5	0.051	0.029	0.004	0.051
4	rs1515110	NR	0.013	0.042	0.027	0.042
5	rs515135	APOB	0.013	0.003	0.041	0.041
6	rs6859	APOE	0.035	0.018	0.039	0.039
7	rs2326077	intergenic	0.039	0.027	0.015	0.039
8	rs5880	CETP	0.001	0.038	0.023	0.038
9	rs799160	intergenic	0.004	0.002	0.037	0.037
10	rs4465830	ZNF335	0.005	0.037	0.037	0.037
		threshold	0.457	0.696	0.457	

CARDIoGRAMplusC4D only

	rs	region	$Cd1$	$Cd2$	$Cd3$	max Cd
1	rs261342	LIPC	0.008	0.024	0.911	**0.911**
2	rs5880	CETP	0.006	0.164	0.057	0.164
3	rs515135	APOB	0.116	0.129	0.125	0.129
4	rs2923084	AMPD3	0.081	0.109	0.096	0.109
5	rs10903129	TMEM57	0.078	0.012	0.025	0.078
6	rs4530754	CSNK1G3	0.076	0.001	0.001	0.076
7	rs6489818	MAPKAPK5	0.062	0.005	0.009	0.062
8	rs2326077	intergenic	0.039	0.016	0.015	0.039
9	rs12133576	DR1	0.036	0.006	0.004	0.036
10	rs4465830	ZNF335	0.005	0.036	0.000	0.036
		threshold	0.457	0.457	0.457	

UK Biobank only

	rs	region	$Cd1$	$Cd2$	$Cd3$	$Cd4$	max Cd
1	rs10401969	SUGP1	0.302	0.224	0.248	0.096	0.302
2	rs2923084	AMPD3	0.124	0.064	0.026	0.079	0.124
3	rs5880	CETP	0.107	0.033	0.025	0	0.107
4	rs2297374	SLC22A1	0.024	0.054	0.091	0.057	0.091
5	rs10903129	TMEM57	0.012	0.051	0.005	0.071	0.071
6	rs7703051	HMGCR	0.006	0.053	0.009	0.065	0.065
7	rs6489818	MAPKAPK5	0.005	0.032	0.001	0.055	0.055
8	rs894210	intergenic	0.05	0.051	0.019	0.039	0.051
9	rs687339	intergenic	0.038	0.044	0.039	0.045	0.045
10	rs998584	VEGFA	0.041	0.039	0.037	0.036	0.041
		threshold	0.457	0.457	0.696	0.457	

Table S3: Influential genetic variants: This table displays for each study the 10 variants with the largest Cook's distance (Cd) and the annotated genomic region based on the best individual models (model posterior probability > 0.02). The maximum Cd of each variant in all models is used for diagnostics. The final row gives the suggested cut-off for Cook's distance and genetic variants with Cd above the threshold are marked in bold.

CARDIoGRAMplusC4D and UK Biobank

	rs	gene region	$q1$	$q2$	$q3$	max q
1	rs1250229	FN1	55.077	54.867	57.211	**57.211**
2	rs6489818	MAPKAPK5	19.308	20.150	12.288	**20.150**
3	rs12801636	PCNX3	15.124	14.625	15.845	**15.845**
4	rs1515110	NR	14.697	10.106	11.196	**14.697**
5	rs2290547	SETD2	13.361	14.316	8.53	**14.316**
6	rs2297374	SLC22A1	11.075	11.676	14.204	**14.204**
7	rs10903129	TMEM57	13.910	12.503	7.369	**13.910**
8	rs2925979	CMIP	13.787	11.646	10.338	**13.787**
9	rs2240327	RBM6	13.213	11.505	11.223	**13.213**
10	rs4465830	ZNF335	8.194	2.964	12.962	**12.962**
11	rs6450176	ARL15	8.271	6.936	12.705	12.705
12	rs731839	PEPD	12.596	10.504	10.14	12.596
13	rs4148218	ABCG8	11.789	12.03	12.032	12.032
14	rs2247056	HLA	8.710	9.897	11.563	11.563
15	rs9930333	FTO	7.213	6.599	11.191	11.191
		threshold				12.84801

CARDIoGRAMplusC4D only

	rs	gene region	$q1$	$q2$	$q3$	max q
1	rs4530754	CSNK1G3	24.505	15.468	15.292	**24.505**
2	rs6489818	MAPKAPK5	19.513	14.598	13.255	**19.513**
3	rs12801636	PCNX3	16.290	16.800	16.810	**16.810**
4	rs4148218	ABCG8	14.936	14.107	15.098	**15.098**
5	rs1250229	FN1	9.932	12.776	10.769	12.776
6	rs952044	AC090771.2	10.333	12.468	11.714	12.468
7	rs2297374	SLC22A1	9.125	9.187	11.492	11.492
8	rs4465830	ZNF335	7.196	5.401	11.390	11.390
9	rs998584	VEGFA	8.781	11.195	7.745	11.195
10	rs2923084	AMPD3	8.404	10.802	9.845	10.802
		threshold				12.84801

UK Biobank only

	rs	gene region	$q1$	$q2$	$q3$	$q4$	max q
1	rs2297374	SLC22A1	38.863	34.587	27.820	34.345	**38.863**
2	rs1250229	FN1	25.528	22.807	31.310	24.057	**31.310**
3	rs6489818	MAPKAPK5	14.222	18.563	12.616	20.625	**20.625**
4	rs2240327	RBM6	15.063	16.844	16.278	17.034	**17.034**
5	rs687339	intergenic	16.284	15.452	7.003	15.328	**16.284**
6	rs2925979	CMIP	10.424	15.160	9.803	13.903	**15.160**
7	rs4148218	ABCG8	14.250	14.512	11.681	14.137	**14.512**
8	rs4921914	NAT2	13.259	10.640	10.262	11.642	**13.259**
9	rs1186380	HNF1A-AS1	9.758	12.067	11.982	13.168	**13.168**
10	rs2241210	UBE3B	12.630	11.045	10.759	9.015	12.630
		threshold					12.87313

Table S4: Outlying genetic variants: This table displays for each study the 10 variants with the largest maximum q and the annotated genomic region based on the best individual models (model posterior probability > 0.02). The maximum q of each variant in all models is used for diagnostics. The final row gives the suggested threshold for the $q-$statistic and variants with q above this threshold are given in bold.

	CARDIoGRAMplusC4D and UK Biobank					
	Model	Posterior probability	Causal effect	Risk factor	Marginal inclusion probability	Model-averaged causal effect
1	ApoB	0.347	0.432	ApoB	0.706	0.298
2	ApoB,M.HDL.C	0.048	0.392,-0.17	M.HDL.C	0.124	-0.024
3	XS.VLDL.TG	0.039	0.411	XS.VLDL.TG	0.103	0.032
4	ApoB,S.LDL.C	0.015	0.613,-0.208	IDL.TG	0.079	0.021
5	ApoB,SM	0.014	0.501,-0.139	XXL.VLDL.TG	0.076	0.02
6	IDL.TG	0.014	0.38	IDL.C	0.074	0.018
7	ApoB,S.HDL.TG	0.014	0.334,0.151	LDL.C	0.052	0.005
8	ApoB,XS.VLDL.TG	0.014	0.287,0.163	Serum.TG	0.049	0.014
9	ApoB,XXL.VLDL.TG	0.013	0.37,0.156	Serum.C	0.048	0.009

	CARDIoGRAMplusC4D only					
	Model	Posterior probability	Causal effect	Risk factor	Marginal inclusion probability	Model-averaged causal effect
1	ApoB	0.24	0.438	ApoB	0.488	0.197
2	XS.VLDL.TG	0.058	0.42	IDL.TG	0.159	0.048
3	IDL.TG	0.033	0.395	XS.VLDL.TG	0.153	0.05
4	S.VLDL.C	0.015	0.447	Serum.TG	0.095	0.036
5	ApoB,XS.VLDL.TG	0.014	0.272,0.186	Tot.FA	0.088	0.026
6	ApoB,S.HDL.TG	0.012	0.331,0.163	IDL.C	0.076	0.016
7	ApoB,IDL.TG	0.012	0.283,0.167	S.HDL.TG	0.07	0.016
8	IDL.TG,XXL.VLDL.	0.012	0.319,0.256	XXL.VLDL.TG	0.067	0.016
9	ApoB,M.HDL.C	0.01	0.407,-0.127	Serum.C	0.065	0.016
10	ApoB,Serum.TG	0.01	0.318,0.16	S.LDL.C	0.064	0.011

	UK Biobank only					
	Model	Posterior probability	Causal effect	Risk factor	Marginal inclusion probability	Model-averaged causal effect
1	XS.VLDL.TG	0.205	0.459	XS.VLDL.TG	0.388	0.161
2	S.VLDL.C	0.032	0.488	Tot.FA	0.321	0.139
3	HDL.C,Tot.FA	0.03	-0.255,0.475	ApoB	0.147	0.047
4	ApoB	0.023	0.452	IDL.TG	0.145	0.045
5	IDL.TG	0.019	0.425	HDL.C	0.103	-0.023
6	ApoB,XS.VLDL.TG	0.014	0.191,0.294	S.VLDL.C	0.099	0.033
7	L.HDL.C,Tot.FA	0.013	-0.221,0.448	S.HDL.TG	0.097	0.026
8	S.HDL.TG,Tot.FA	0.011	0.329,0.259	TotPG	0.089	-0.032
9	Tot.FA,TotPG	0.01	0.883,-0.504	IDL.C	0.073	0.015
10	LDL.C,XS.VLDL.TG	0.009	0.129,0.369	Serum.TG	0.072	0.026

Table S5: Analysis including all genetic variants: Top 10 models ranked by the model posterior probability and top 10 risk factors ranked by the marginal inclusion probability including all genetic variants before removing influential genetic variants and outliers. Causal effects are log odds ratios for coronary artery disease per 1 standard deviation increase in the risk factor.

	CARDIoGRAMplusC4D and UK Biobank					
	Model	Posterior probability	Causal effect	Risk factor	Marginal inclusion probability	Model-averaged causal effect
1	ApoB	0.472	0.46	ApoB	0.862	0.385
2	ApoB,S.HDL.TG	0.043	0.343,0.177	S.HDL.TG	0.136	0.033
3	LDL.C,S.HDL.TG	0.02	0.272,0.301	LDL.C	0.076	0.015
4	ApoB,M.HDL.C	0.019	0.435,-0.11	XXL.VLDL.TG	0.05	0.011
5	ApoB,XXL.VLDL.TG	0.015	0.408,0.123	Serum.C	0.045	0.01
6	ApoB,S.LDL.C	0.015	0.571,-0.127	IDL.C	0.043	0.008
7	ApoB,XS.VLDL.TG	0.012	0.367,0.102	S.LDL.C	0.041	0.001
8	ApoB,Serum.TG	0.011	0.385,0.098	Serum.TG	0.038	0.007
9	ApoB,LDL.C	0.011	0.525,-0.071	M.HDL.C	0.037	-0.004
10	ApoB,S.VLDL.C	0.011	0.474,-0.015	HDL.C	0.035	-0.005

Table S6: Supplementary analysis 1: After excluding the genetic variant in the *APOB* gene region, these are the top 10 models judged by posterior probability and top 10 risk factors judged by marginal inclusion probability in the primary analysis based on $n = 137$ genetic variants. Causal effects are log odds ratios for coronary artery disease per 1 standard deviation increase in the risk factor.

	Model	Posterior probability	Causal effect	Risk factor	Marginal inclusion probability	Model-averaged causal effect	Empirical $p-$value	FDR
CARDIoGRAMplusC4D and UK Biobank including all genetic variants (n = 148)								
1	XS.VLDL.TG	0.127	0.411	XS.VLDL.TG	0.263	0.094		
2	IDL.TG	0.046	0.38	IDL.C	0.213	0.059		
3	S.VLDL.C	0.041	0.44	IDL.TG	0.204	0.063		
4	IDL.C,XXL.VLDL.TG	0.03	0.299,0.347	XXL.VLDL.TG	0.168	0.05		
5	IDL.TG,XXL.VLDL.TG	0.022	0.304,0.267	M.HDL.C	0.162	-0.037		
6	M.HDL.C,Serum.C	0.015	-0.317,0.367	Serum.C	0.116	0.034		
7	LDL.C,XS.VLDL.TG	0.015	0.178,0.286	LDL.C	0.114	0.026		
8	IDL.C,S.HDL.TG	0.012	0.241,0.282	Serum.TG	0.107	0.038		
9	IDL.C,Serum.TG	0.011	0.219,0.287	S.VLDL.C	0.096	0.031		
10	S.LDL.C,XS.VLDL.TG	0.011	0.175,0.294	S.HDL.TG	0.079	0.019		
CARDIoGRAMplusC4D and UK Biobank after model diagnostics (n = 138)								
	Model	Posterior probability	Causal effect	Risk factor	Marginal inclusion probability	Model-averaged causal effect	Empirical $p-$value	FDR
1	LDL.C,S.HDL.TG	0.156	0.261,0.3	S.HDL.TG	0.461	0.144	0.0021	0.025
2	IDL.TG	0.063	0.436	LDL.C	0.417	0.119	0.0013	0.025
3	S.HDL.TG,S.LDL.C	0.049	0.294,0.266	Serum.C	0.17	0.056	0.0143	0.087
4	IDL.C,S.HDL.TG	0.048	0.237,0.325	IDL.TG	0.159	0.055	0.0151	0.087
5	L.HDL.C,Serum.C	0.032	-0.272,0.381	S.LDL.C	0.156	0.038	0.0274	0.101
6	HDL.C,Serum.C	0.027	-0.277,0.441	IDL.C	0.128	0.029	0.0296	0.101
7	S.HDL.TG,Serum.C	0.021	0.354,0.23	L.HDL.C	0.118	-0.026	0.0181	0.087
8	LDL.C,XS.VLDL.TG	0.016	0.233,0.249	HDL.C	0.095	-0.019	0.0384	0.115
9	Est.C,S.HDL.TG	0.014	0.197,0.393	XS.VLDL.TG	0.076	0.017	0.0682	0.182
10	LDL.C,XXL.VLDL.TG	0.012	0.337,0.273	XXL.VLDL.TG	0.073	0.016	0.2636	0.575

Table S7: Supplementary analysis 2: After excluding the ApoB measurement as risk factor from the set of candidate risk factors these are the top 10 models ranked by the posterior probability and top 10 risk factors ranked by the marginal inclusion probability in the primary analysis based on all available genetic variants (n = 148) and after model diagnostics (n = 138). Causal effects are log odds ratios for coronary artery disease per 1 standard deviation increase in the risk factor.

CARDIoGRAMplusC4D and UK Biobank including all NMR GWAS genetic variants (n = 55)

	Model	Posterior probability	Causal effect	Risk factor	Marginal inclusion probability	Model-averaged causal effect
1	M.VLDL.C,XXL.VLDL.TG	0.063	0.957,-1.018	M.VLDL.C	0.325	0.312
2	IDL.TG	0.061	0.397	XXL.VLDL.TG	0.32	-0.267
3	Serum.TG,XXL.VLDL.TG	0.046	0.974,-1.025	Serum.TG	0.214	0.19
4	L.VLDL.C,M.VLDL.C	0.038	-1.113,1.224	XL.VLDL.TG	0.173	-0.13
5	M.VLDL.C,XL.VLDL.TG	0.036	0.966,-0.961	IDL.TG	0.149	0.057
6	IDL.C	0.036	0.348	L.VLDL.C	0.134	-0.107
7	ApoB	0.023	0.369	L.VLDL.TG	0.115	-0.083
8	Serum.TG,XL.VLDL.TG	0.02	0.974,-0.954	IDL.C	0.1	0.033
9	L.VLDL.TG,Serum.TG	0.016	-0.998,1.065	S.VLDL.C	0.095	0.044
10	S.VLDL.C,XXL.VLDL.TG	0.013	0.565,-0.385	XS.VLDL.TG	0.068	0.029

CARDIoGRAMplusC4D and UK Biobank after model diagnostics (n = 45)

	Model	Posterior probability	Causal effect	Risk factor	Marginal inclusion probability	Model-averaged causal effect	Empirical $p-$value	FDR
1	ApoB	0.155	0.3	ApoB	0.492	0.153	1.0E-04	0.004
2	S.VLDL.C	0.077	0.269	S.VLDL.C	0.246	0.068	4.0E-04	0.009
3	ApoB,Crea	0.047	0.318,-0.196	Crea	0.241	-0.049	0.026	0.247
4	Crea,S.VLDL.C	0.025	-0.201,0.287	M.VLDL.C	0.07	0.022	0.013	0.201
5	XS.VLDL.TG	0.015	0.227	S.LDL.C	0.069	0.014	0.030	0.247
6	ApoB,Gly	0.014	0.303,-0.064	Phe	0.067	-0.011	0.137	0.441
7	ApoB,Phe	0.011	0.306,-0.157	Gly	0.067	-0.004	0.043	0.276
8	S.LDL.C	0.01	0.329	XS.VLDL.TG	0.062	0.01	0.033	0.247
9	M.VLDL.C	0.008	0.225	S.HDL.TG	0.06	0.011	0.072	0.404
10	S.HDL.TG	0.007	0.244	M.HDL.C	0.06	-0.01	0.117	0.441

Table S8: Supplementary analysis 3: We additionally varied our set of instruments and performed an analysis based on genetic variants associated with any of the measures in the metabolomics genome-wide association study by Kettunen et al. These are the top 10 models ranked by the posterior probability and top 10 risk factors ranked by the marginal inclusion probability in the primary analysis based on all available genetic variants (n = 55) and after model diagnostics (n = 45). Causal effects are log odds ratios for coronary artery disease per 1 standard deviation increase in the risk factor.

CARDIoGRAMplusC4D only

	Model	Posterior probability	Causal effect	Risk factor	Marginal inclusion probability	Model-averaged causal effect	Empirical $p-$value	FDR
1	ApoB	0.394	0.455	ApoB	0.673	0.293	0.0001	0.003
2	ApoB,M.HDL.C	0.018	0.425,-0.121	LDL.C	0.107	0.027	0.0544	0.461
3	S.VLDL.C	0.018	0.464	S.LDL.C	0.097	0.027	0.0709	0.461
4	IDL.TG	0.014	0.444	Serum.TG	0.084	0.028	0.0599	0.461
5	HDL.C,Serum.C	0.014	-0.263,0.464	Serum.C	0.072	0.021	0.1176	0.510
6	LDL.C,Serum.TG	0.012	0.276,0.263	HDL.C	0.062	-0.012	0.0974	0.506
7	ApoB,Serum.TG	0.011	0.369,0.115	S.VLDL.C	0.059	0.015	0.1667	0.542
8	ApoB,IDL.TG	0.011	0.358,0.109	IDL.TG	0.056	0.015	0.1539	0.542
9	S.LDL.C	0.010	0.461	M.HDL.C	0.055	-0.008	0.1889	0.546
10	ApoB,S.VLDL.C	0.010	0.402,0.06	IDL.C	0.052	0.010	0.2423	0.630

UK Biobank only

	Model	Posterior probability	Causal effect	Risk factor	Marginal inclusion probability	Model-averaged causal effect	Empirical $p-$value	FDR
1	XS.VLDL.TG	0.195	0.435	XS.VLDL.TG	0.456	0.169	0.0002	0.006
2	ApoB,S.HDL.TG	0.056	0.281,0.233	ApoB	0.325	0.102	0.0010	0.015
3	ApoB,XS.VLDL.TG	0.043	0.207,0.258	S.HDL.TG	0.222	0.060	0.0061	0.061
4	ApoB	0.039	0.437	IDL.TG	0.109	0.027	0.0157	0.103
5	LDL.C,XS.VLDL.TG	0.024	0.14,0.338	LDL.C	0.108	0.018	0.0446	0.191
6	S.VLDL.C	0.024	0.467	Serum.TG	0.104	0.032	0.0171	0.103
7	LDL.C,S.HDL.TG	0.015	0.216,0.334	S.VLDL.C	0.086	0.024	0.0444	0.191
8	S.LDL.C,XS.VLDL.TG	0.015	0.133,0.346	Tot.FA	0.079	0.018	0.0677	0.254
9	IDL.C,S.HDL.TG	0.012	0.201,0.345	IDL.C	0.063	0.009	0.0994	0.331
10	ApoB,Serum.TG	0.012	0.273,0.218	S.LDL.C	0.059	0.003	0.1739	0.522

Table S9: Sensitivity analysis: Top 10 models ranked by the posterior probability and top 10 risk factors ranked by the marginal inclusion probability after model diagnostics (including n = 144 genetic variants for CARDIoGRAMplusC4D and n = 141 for UK Biobank). Causal effects are log odds ratios for coronary artery disease per 1 standard deviation increase in the risk factor.

σ = 0.1

#	risk factor	*MIP*	$\hat{\theta}_{\mathrm{MACE}}$
1	ApoB	0.638	0.193
2	S.HDL.TG	0.365	0.073
3	LDL.C	0.253	0.05
4	IDL.C	0.165	0.029
5	IDL.TG	0.14	0.019
6	XXL.VLDL.TG	0.134	0.019
7	XS.VLDL.TG	0.127	0.018
8	Serum.TG	0.117	0.016
9	Serum.C	0.115	0.019
10	M.HDL.C	0.11	-0.013

σ = 0.2

#	risk factor	*MIP*	$\hat{\theta}_{\mathrm{MACE}}$
1	ApoB	0.759	0.307
2	S.HDL.TG	0.261	0.061
3	LDL.C	0.144	0.031
4	XXL.VLDL.TG	0.099	0.018
5	IDL.C	0.091	0.018
6	Serum.C	0.076	0.016
7	M.HDL.C	0.075	-0.009
8	Serum.TG	0.071	0.011
9	S.LDL.C	0.064	0.007
10	XS.VLDL.TG	0.064	0.008

σ = 0.3

#	risk factor	*MIP*	$\hat{\theta}_{\mathrm{MACE}}$
1	ApoB	0.818	0.355
2	S.HDL.TG	0.201	0.048
3	LDL.C	0.105	0.022
4	XXL.VLDL.TG	0.072	0.014
5	IDL.C	0.064	0.012
6	Serum.C	0.061	0.014
7	M.HDL.C	0.057	-0.007
8	Serum.TG	0.051	0.008
9	HDL.C	0.048	-0.007
10	S.LDL.C	0.048	0.003

σ = 0.5

#	risk factor	*MIP*	$\hat{\theta}_{\mathrm{MACE}}$
1	ApoB	0.868	0.392
2	S.HDL.TG	0.136	0.033
3	LDL.C	0.075	0.015
4	XXL.VLDL.TG	0.047	0.01
5	Serum.C	0.045	0.011
6	IDL.C	0.042	0.008
7	S.LDL.C	0.04	0.001
8	M.HDL.C	0.038	-0.005
9	HDL.C	0.036	-0.006
10	Serum.TG	0.035	0.006

σ = 0.7

#	risk factor	*MIP*	$\hat{\theta}_{\mathrm{MACE}}$
1	ApoB	0.907	0.415
2	S.HDL.TG	0.101	0.025
3	LDL.C	0.055	0.011
4	XXL.VLDL.TG	0.03	0.006
5	S.LDL.C	0.029	0
6	Serum.C	0.029	0.006
7	IDL.C	0.028	0.005
8	M.HDL.C	0.026	-0.003
9	Serum.TG	0.023	0.004
10	HDL.C	0.023	-0.003

Table S10: Parameter check for the prior variance σ^2, ranging from σ = 0.1 to σ = 0.7. The main analysis used σ = 0.5. Abbreviations: *MIP*=marginal inclusion probability, MACE=model-averaged causal effect.

$p = 0.01$			
#	risk factor	*MIP*	$\hat{\theta}_{\text{MACE}}$
1	ApoB	0.979	0.454
2	S.HDL.TG	0.015	0.004
3	LDL.C	0.009	0.002
4	S.VLDL.C	0.007	0.002
5	S.LDL.C	0.004	0
6	Serum.C	0.004	0.001
7	XS.VLDL.TG	0.004	0.001
8	IDL.C	0.004	0.001
9	M.HDL.C	0.004	0
10	XXL.VLDL.TG	0.004	0.001

$p = 0.05$			
#	risk factor	*MIP*	$\hat{\theta}_{\text{MACE}}$
1	ApoB	0.929	0.426
2	S.HDL.TG	0.071	0.017
3	LDL.C	0.039	0.008
4	Serum.C	0.022	0.005
5	S.LDL.C	0.02	0.001
6	XXL.VLDL.TG	0.02	0.004
7	IDL.C	0.02	0.004
8	M.HDL.C	0.019	-0.002
9	HDL.C	0.017	-0.003
10	S.VLDL.C	0.016	0.001

$p = 0.1$			
#	risk factor	*MIP*	$\hat{\theta}_{\text{MACE}}$
1	ApoB	0.868	0.392
2	S.HDL.TG	0.136	0.033
3	LDL.C	0.075	0.015
4	XXL.VLDL.TG	0.047	0.01
5	Serum.C	0.045	0.011
6	IDL.C	0.042	0.008
7	S.LDL.C	0.04	0.001
8	M.HDL.C	0.038	-0.005
9	HDL.C	0.036	-0.006
10	Serum.TG	0.035	0.006

$p = 0.2$			
#	risk factor	*MIP*	$\hat{\theta}_{\text{MACE}}$
1	ApoB	0.791	0.347
2	S.HDL.TG	0.238	0.059
3	LDL.C	0.127	0.025
4	XXL.VLDL.TG	0.099	0.022
5	Serum.C	0.083	0.019
6	IDL.C	0.076	0.013
7	Serum.TG	0.071	0.014
8	S.LDL.C	0.07	0.001
9	M.HDL.C	0.068	-0.008
10	HDL.C	0.068	-0.01

$p = 0.3$			
#	risk factor	*MIP*	$\hat{\theta}_{\text{MACE}}$
1	ApoB	0.744	0.318
2	S.HDL.TG	0.32	0.081
3	XXL.VLDL.TG	0.18	0.046
4	LDL.C	0.164	0.032
5	Serum.TG	0.117	0.025
6	Serum.C	0.11	0.023
7	IDL.C	0.106	0.018
8	S.VLDL.C	0.103	-0.014
9	M.HDL.C	0.092	-0.011
10	S.LDL.C	0.092	0.001

Table S11: Parameter check for the prior probability p, ranging from $p = 0.01$ to $p = 0.3$. This reflects 0.3 to 9 expected causal risk factors. The main analysis used $p = 0.1$ reflecting an a priori expected number of 3 causal risk factors. Abbreviations: *MIP*=marginal inclusion probability, MACE=model-averaged causal effect.

Supplementary Figures

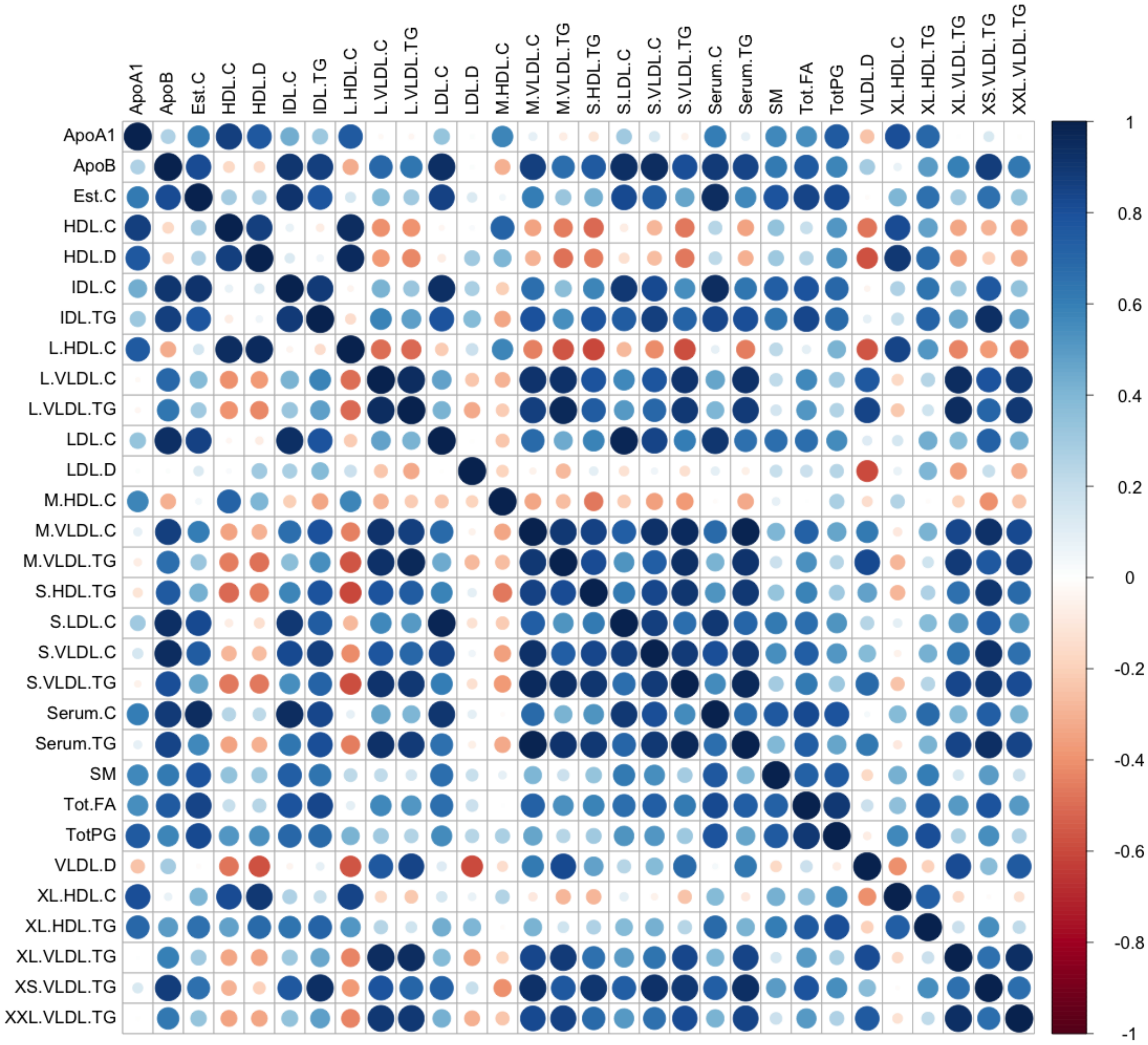

Figure S1: Genetic correlation between lipoprotein measures and metabolites based on the n = 148 lipid-associated genetic variants as used in the main analysis. Colorcode indicates correlation strength (darkblue=strong positive correlation to darkred=strong negative correlation). The size of the square is proportional to the absolute correlation.

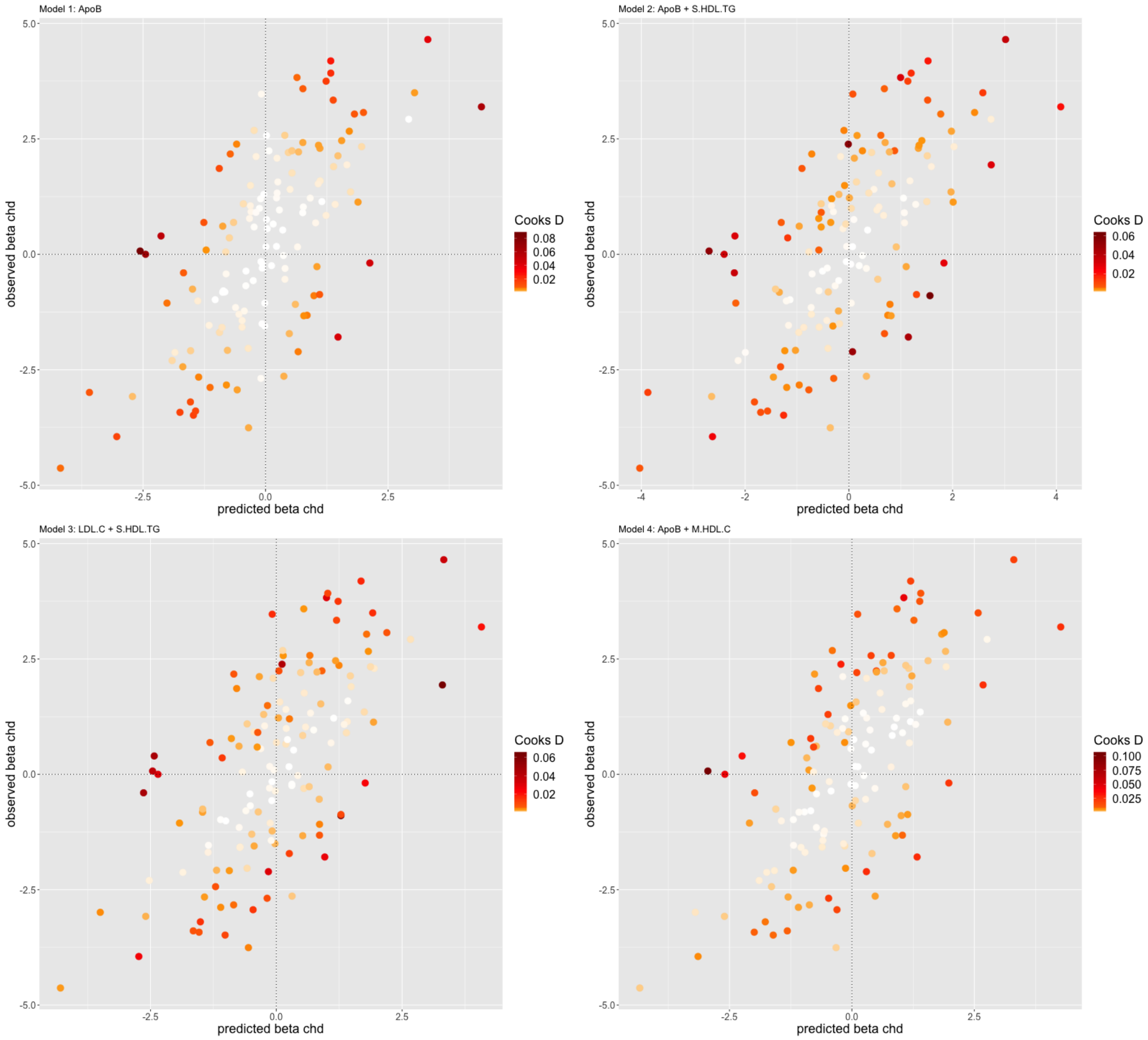

Figure S2: Diagnostic plots with Cooks distance: Estimates of genetic associations with the outcome against predicted genetic associations with the outcome from the primary analysis based on $n = 138$ genetic variants after exclusion of outliers. Here we show the diagnostics for all four top models with posterior probability > 0.02 as given in Main Table 1.Colour code of points indicates influence, as measured by the variant's Cook's distance.

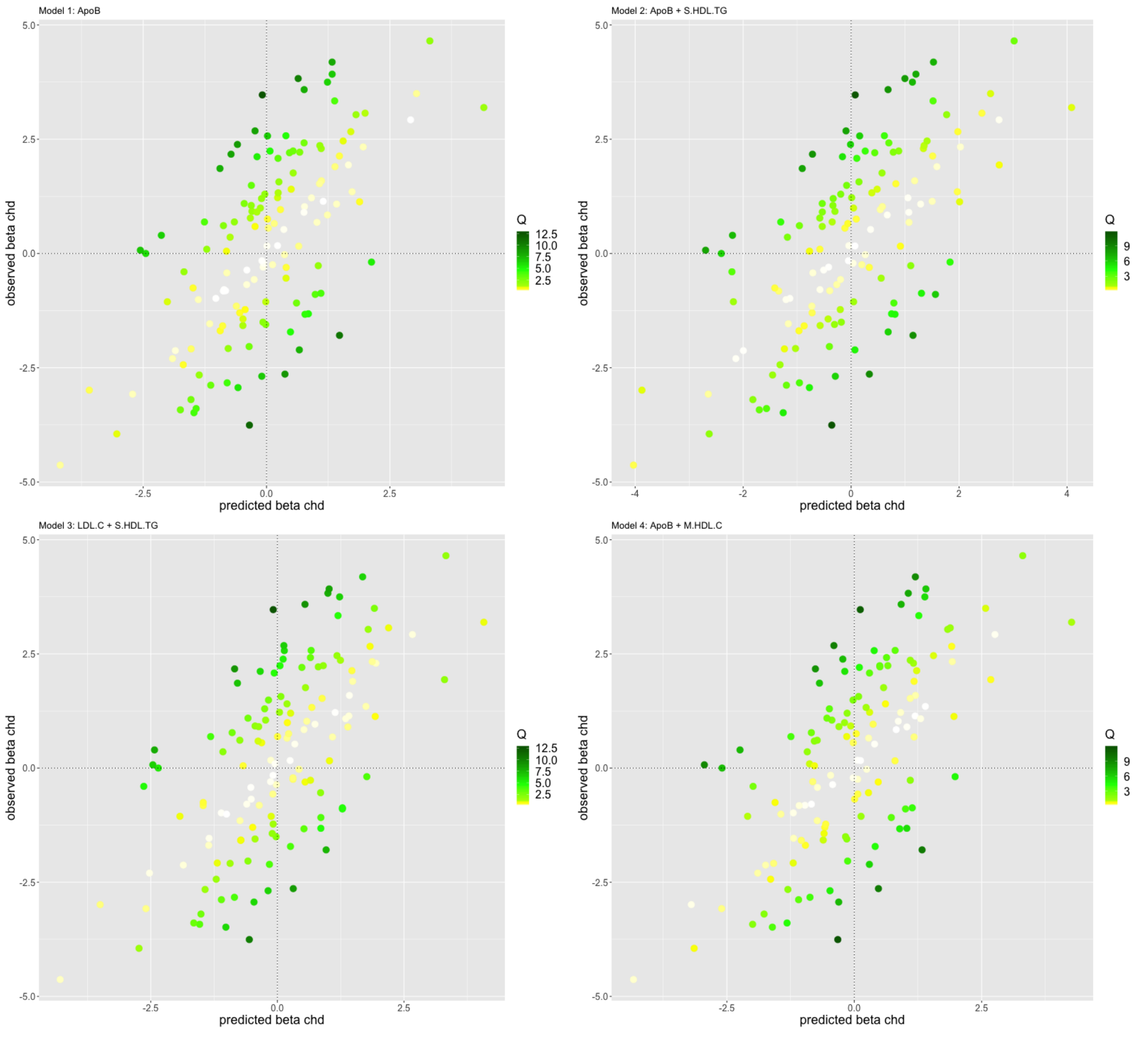

Figure S3: Diagnostic plots with q-statistic: Estimates of genetic associations with the outcome against predicted genetic associations with the outcome from the primary analysis based on $n = 138$ genetic variants after exclusion of outliers. Here we show the diagnostics for all four top models with posterior probability > 0.02 as given in Main Table 1. Colour code of points indicates heterogeneity, as measured by the variant's q-statistic.

Supplementary Methods

Mendelian randomization using summarized data

A genetic variant can be used to make causal inferences about the effect of a risk factor on an outcome if it satisfies the three instrumental variable assumptions:

IV1 The variant is associated with the risk factor;

IV2 The variant is not confounded in its associations with the outcome;

IV3 The variant does not influence the outcome directly, only potentially indirectly via its association with the risk factor.

These assumptions imply that a genetic variant behaves analogously to random assignment to a treatment group in a randomized controlled trial, in that it divides the population into subgroups that differ only with respect to their average level of the risk factor [1]. Any difference in the outcome between these groups implies a causal effect of the risk factor on the outcome, analogous to an intention-to-treat effect in a randomized trial [2].

We consider an extension of the Mendelian randomization paradigm known as multivariable Mendelian randomization, in which genetic variants are allowed to influence multiple risk factors, provided that any causal pathway from the genetic variants to the outcome passes via one or more of the measured risk factors [3]. The assumptions for genetic variants to be valid instruments in multivariable Mendelian randomization are:

MV-IV1 Each variant is associated with at least one of the risk factors;

MV-IV2 Variants are not confounded in their associations with the outcome;

MV-IV3 Variants are not associated with the outcome conditional on the risk factors and confounders.

In turn, the assumptions for a risk factor to be included in a multivariable Mendelian randomization model are:

RF1 No risk factor can be linearly explained by any other included risk factor or a combination of multiple risk factors.

RF2 Each risk factor is associated with at least one of the genetic variants.

Assumption RF1 is needed to distinguish between correlated risk factors [4]. RF2 ensures that each risk factor is adequately predicted by the genetic variants selected as instrumental variables in the analysis.

For a particular set of risk factors, causal effects are estimated by weighted linear regression of the genetic associations with the outcome on the genetic associations with the risk factors

$$\beta_Y = \theta_1\beta_{X1} + \theta_2\beta_{X2} + \ldots + \theta_d\beta_{Xd} + \varepsilon, \quad \varepsilon \sim N(0, \text{diag}(\text{se}(\beta_Y)^2)),$$

where β_Y is the vector of genetic associations with the outcome of length n, with n the number of genetic variants used as instrumental variables, $\text{se}(\beta_Y)$ is the vector of standard errors of these associations of length n and diag the diagonal operator. $\beta_{X1}, \beta_{X2}, \ldots, \beta_{Xd}$ are the genetic associations with the d risk factors, and $\theta_1, \theta_2, \ldots, \theta_d$ are the causal effects of the d risk factors on the outcome. If there are causal relationships between the risk factors, then these parameters represent the direct effects of the risk factors, i.e. the effect of changing the target risk factor keeping all other risk factors constant [4, 5].

Variable selection and Bayesian model averaging

The model averaging approach is implemented by considering different sets of risk factors in turn [6]. For each risk factor set, MR-BMA fits the relevant multivariable Mendelian randomization model and assigns a score to the set of risk factors considered that captures the posterior probability that this particular model represents the true causal risk factors for the outcome given the observed genetic association data [6]. As prior parameters MR-BMA requires to set an a priori probability for a risk factor to be causal, which is set to 0.1 reflecting an a priori epectation of three causal risk factors. Additionally, the prior variance is set to 0.25. Sensitivity analysis with respect to the prior parameters is important and we can show that ranking is not impacted by the choice of the prior. Results for a wide range of prior specifications are given in Supplementary Table S10 (prior variance) and Supplementary Table S11 (prior probability).

When considering many candidate risk factors, the model space (including all possible combinations of risk factors) may be prohibitively large to consider all possible combinations of risk factors. To alleviate this we have implemented a stochastic search algorithm [7] to explore the relevant model space (all models with a non-negligible posterior probability) in an efficient way.

When the number of risk factors considered is large, the evidence for each particular model may be small. Hence, we average over the models visited and for each risk factor compute its marginal inclusion probability, which is the sum of the posterior probabilities for all models visited that include this particular risk factor. Further, we provide the model-averaged causal effect estimate, representing the average causal effect estimate for the given risk factor across models in which it is included. As is common for variable-selection methods, this is a conservative estimates of the true causal effect and underestimates its magnitude, but may be used for the interpretation of effect direction and for comparison among the risk factors.

Resampling to compute empirical $p-$values

Empirical p-values for the marginal inclusion probability of each risk factor are obtained using a permutation procedure, where the risk factor association data are held constant and the outcome associations of the genetic variants are randomly perturbed [8]. The empirical p-value for risk factor j quantifies how extreme the actual observed marginal inclusion probability is with respect to all permuted marginal inclusion probabilities for that particular risk factor. Formally, the empirical p-value is computed by the rank (r_j) of the actual observed marginal inclusion probability for risk factor j among all permuted marginal inclusion probabilities for risk factor j over the total number of permutations (n_{perm} = 1,000). Following [9] we add one to the computation to obtain the probability that under the null hypothesis the observed marginal inclusion probability has the observed or a higher rank

$$p_j = (r_j + 1)/(n_{perm} + 1).$$

Multiple testing adjustment is done using the Benjamini and Hochberg false discovery rate (FDR) procedure [10].

Model diagnostics

Two approaches are considered for model diagnostics. Firstly, to identify influential variants for each visited model with a model posterior probability larger than 0.02, we calculated Cook's distance for each genetic variant [11] and excluded all variants that have in any selected model a Cook's distance which exceeds the median of a central F-distribution with d and $n-d$ degrees of freedom, where d is the number of risk factors and n the number of genetic variants used as instrumental variables.

Secondly, to identify outlying variants, we consider for each visited model with a model posterior probability larger than 0.02 a version of Cochran's Q statistic used to detect heterogeneity in meta-analysis [12]

$$Q = \sum_{i=1}^{n} q_i = \sum_{i=1}^{n} \mathrm{se}(\beta_{Y_i})^{-2}(\beta_{Y_i} - \hat{\beta}_{Y_i})^2,$$

where i indexes the genetic variants and $\hat{\beta}_{Y_i}$ is the predicted value of the genetic association with the outcome β_{Y_i} based on the relevant multivariable Mendelian randomization model. A genetic variant with a high value of q_i (compared to the $0.05/n$th upper tail of a χ^2 distribution with one degree of freedom representing Bonferroni multiple testing adjustment by the number of variants included) in any of the models visited (with a model posterior probability larger than 0.02) was considered to be an outlying variant.

We then repeated the analyses excluding such variants. The reason for excluding outliers and influential variants is that a single genetic variant can have a strong impact on the models visited and subsequently on variable selection. However, in this case for both main and sensitivity analyses, excluding these variants did not change the headline results.

References

[1] Thanassoulis G, O'Donnell CJ. Mendelian randomization: nature's randomized trial in the post-genome era. JAMA. 2009;301(22):2386–2388.

[2] Burgess S, Foley CN, Zuber V. Inferring causal relationships between risk factors and outcomes from genome-wide association study data. Annu Rev Genomics Hum Genet. 2018;.

[3] Burgess S, Thompson SG. Multivariable Mendelian randomization: the use of pleiotropic genetic variants to estimate causal effects. Am. J. Epidemiol. 2015;181(4):251–260.

[4] Sanderson E, Davey Smith G, Windmeijer F, Bowden J. An examination of multivariable Mendelian Randomization in the single-sample and two-sample summary data settings. Int J Epidemiol. 2018 12;p. dyy262–dyy262.

[5] Burgess S, Thompson DJ, Rees JM, Day FR, Perry JR, Ong KK. Dissecting causal pathways using Mendelian randomization with summarized genetic data: application to age at menarche and risk of breast cancer. Genetics. 2017;.

[6] Zuber V, Colijn JM, Klaver C, Burgess S. Selecting likely causal risk factors from high-throughput experiments using multivariable Mendelian randomization. Nat Commun. 2020;11(1):29.

[7] Hans C, Dobra A, West M. Shotgun stochastic search for "large p" regression. J Am Stat Assoc. 2007;102(478):507–516.

[8] Stephens M, Balding DJ. Bayesian statistical methods for genetic association studies. Nat Rev Genet. 2009 Oct;10(10):681–690.

[9] North BV, Curtis D, Sham PC. A note on the calculation of empirical P values from Monte Carlo procedures. Am. J. Hum. Genet. 2002 08;71(2):439–441.

[10] Benjamini Y, Hochberg Y. Controlling the False Discovery Rate: A Practical and Powerful Approach to Multiple Testing. J R Stat Soc Series B Stat Methodol. 1995;57(1):289–300.

[11] Corbin LJ, Richmond RC, Wade KH, et al. Body mass index as a modifiable risk factor for type 2 diabetes: Refining and understanding causal estimates using Mendelian randomisation. Diabetes. 2016;.

[12] Greco M, Minelli C, Sheehan NA, Thompson JR. Detecting pleiotropy in Mendelian randomisation studies with summary data and a continuous outcome. Stat Med. 2015;34(21):2926–2940.
